# Deciphering the Contribution of Circular RNAs to Age‐Related Decline in Sertoli Cell Survivor

**DOI:** 10.1111/acel.70023

**Published:** 2025-04-18

**Authors:** Francesco Manfrevola, Nicola Mosca, Vincenza Grazia Mele, Teresa Chioccarelli, Guillaume Martinez, Charles Coutton, Monica Mattia, Mariaceleste Pezzullo, Silvia Fasano, Gilda Cobellis, Nicoletta Potenza, Rosanna Chianese

**Affiliations:** ^1^ Department of Experimental Medicine University of Campania L. Vanvitelli Naples Italy; ^2^ Department of Environmental, Biological, Pharmaceutical Sciences and Technologies University of Campania “Luigi Vanvitelli” Caserta Italy; ^3^ Hôpital Couple‐Enfant, Centre Hospitalier Universitaire de Grenoble UM de Génétique Chromosomique Grenoble France; ^4^ Genetic Epigenetic and Therapies of Infertility, Institute for Advanced Biosciences INSERM U1209, CNRS UMR5309 Grenoble France

**Keywords:** age‐related male reproductive decline, ceRNET, circRNAs, FUS protein, male infertility, miR‐214‐3p, Sertoli cell survivor

## Abstract

Male fertility declines during aging. This process mainly affects spermatogonia and Sertoli cells, leading to impaired spermatogenesis and poor‐quality sperm production. Circular RNAs (circRNAs) are covalently closed RNA molecules produced by backsplicing. In the field of male reproduction, circRNAs boast great potential in the regulation of spermatogenesis and sperm morpho‐functional skills. However, their potential role in age‐related male reproductive anomalies remains largely elusive. Here, we analyzed the reproductive phenotype of the aged male mouse experimental model, pointing our attention to a putative functional link between circRNAs and Sertoli cell survival. Our results confirm several testicular age‐related defects including: (i) altered morphology of the seminiferous epithelium; (ii) affected spermatogenesis; and (iii) decreased sperm production. In particular, aged spermatozoa (SPZ) were decreased in number in association with low motility and abnormal morphology (sperm head anomalies and tail bents). The expression analysis of selective spermatic circRNAs demonstrated a de‐regulated expression profile in Aged versus Young SPZ. Among them, we turned the lens on circAbcb9 as a spermatic circRNA potentially involved in the Sertoli cell senescence pathway via the circRNA/miRNA/mRNA network (ceRNET). Indeed, a significant shutdown of circAbcb9‐dependent network associated with a prominent increase in Sertoli cell senescence occurred in Aged testis. Interestingly, circAbcb9 was also expressed in human SPZ at decreased levels in Aged men, suggesting a conserved role. Collectively, our study stimulates greater interest in circRNAs as involved in the molecular mechanisms behind the age‐related effect on Sertoli cell survival, also providing new implications for fused protein in sarcoma (FUS) protein in sertolian circRNA biogenesis.

## Graphical abstract

Sertoli cell survival is regulated by circAbcb9‐ceRNET. Physiologically, the circAbcb9 sponge activity toward miR‐214‐3p ensures the expression of SOX8 and NOTCH2 targets, both crucial in maintaining SC homeostasis. The aging process negatively affects FUS‐dependent backsplicing activity in Sertoli cells leading to circAbcb9 downregulation, and in turn, to the impairment of SOX8 and NOTCH2 targets. These events promote age‐related Sertoli cell senescence and male fertility decline as a consequence.

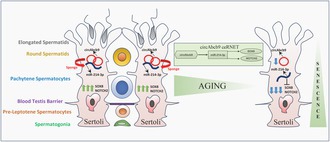

**Abbreviations**
A‐SPZ: high‐quality sperm fraction
B‐SPZ: low‐quality sperm fraction
ceRNET: circRNA/miRNA/mRNA network
circRNAs: circular RNAs
DE‐circRNAs: differentially expressed circRNAs
FUS: fused protein in sarcoma
H&E: hematoxylin and eosin
MTT: 3‐(4,5‐dimethylthiazol‐2‐yl)‐2,5‐diphenyltetrazolium bromide
ncRNAs: non‐coding RNAs
nfe: normalized fold expression
PNA: peanut agglutinin
QKI: quaking protein
RBPs: RNA‐binding proteins
RIP: RNA‐binding protein immunoprecipitation assay
RNApol2: RNA polymerase II
RT‐qPCR: quantitative RT‐PCR
SCLB: Somatic cell lysis buffer
SCOS: Sertoli‐only cell syndrome
SCs: Sertoli cells
SDS‐PAGE: Sodium dodecyl sulfate‐polyacrylamide gel electrophoresis
SPZ: spermatozoa

## 1 Introduction

Testis is the microenvironment in which the spermatogenesis process occurs to ensure lifelong spermatozoa (SPZ) production (O'Donnell et al. 2017). The physiological reproductive decline in male fertility due to aging has negative implications for spermatogenesis, which, in turn, impairs sperm quality parameters in terms of count and morpho‐functional skills, downstream compromising the ability to successfully achieve a pregnancy and increasing the risk of paternal age‐dependent congenital pathology onset (Almeida et al. 2017; Khandwala et al. 2018; Dong et al. 2022; Aitken et al. 2020). Considering that in the last decades the modern societal lifestyles have induced a delay in parenthood, there is currently a growing concern regarding the assessment of age‐related reproductive anomalies, both in terms of mechanisms underlying testicular defects and poor‐quality SPZ, able to negatively impact embryo development and offspring health (Eskenazi et al. 2003; Mills et al. 2011; Kovac et al. 2013; Begueria et al. 2014).

Age‐related spermatogenesis defects include a wide range of anomalies related to spermatogonia and spermatocyte homeostasis, as well as to the activities of somatic Sertoli (SCs) and Leydig cells. Indeed, during physiological aging, spermatogonia undergo a decrease in cell number, dependent on the reduction of cell proliferative activity, which is molecularly associated with nuclear DNA fragmentation (Morales et al. 2003; Pohl et al. 2019). Additionally, aging induces spermatocyte apoptosis, altering oxidative stress pathways (Kokkinaki et al. 2010; Vrooman et al. 2014; Endo et al. 2024) . All together, these testicular defects lead to the final production of SPZ characterized by: (i) an increased DNA fragmentation index; (ii) chromosomal defects associated with premature germ line senescence; and (iii) decreased sperm viability and motility (Mohammadi et al. 2013; Belloc et al. 2014; Kotarska et al. 2017; Shi et al. 2018; Evenson et al. 2020).

Proper maturation of germ cells into fully functional SPZ requires both structural and functional support from the somatic cells forming part of the testicular parenchyma. This parenchyma is composed of two main compartments: (i) convoluted seminiferous tubules, characterized by the presence of germ cells and SCs, and (ii) the surrounding interstitial compartment, mainly composed of vascular and Leydig cells (Kaur et al. 2014). The activities of the parenchymal somatic cells synergistically converge to support spermatogenesis. Indeed, SCs, located on the basement membrane of the seminiferous tubule, provide structural support and supply essential nutrients to developing germ cells via cell–cell contacts; they also secrete several factors involved in the maintenance of the extracellular microenvironment, whereas Leydig cells promote spermatogenesis progression by secreting androgens (Kaur et al. 2014; Heinrich and De Falco 2020; O'Donnell et al. 2022). Although age‐related dysfunctions have been reported for both somatic cell types, several studies have highlighted the propensity of SCs to behave as the cell type most susceptible to testicular aging in terms of premature cellular senescence able to induce spermatogenic failure (Petersen and Soder 2006; Nie et al. 2022; Huang et al. 2023; Xiao et al. 2024).

Interestingly, recent years have seen the emergence of a new fascinating role for non‐coding RNAs (ncRNAs) in age‐related SC dysfunctions, with particular attention on microRNAs involved in SC apoptosis and senescence. These include: (i) miR‐143‐3p, found to be able to promote SC senescence; (ii) miR‐638 and miR‐202‐3p, involved in SC apoptosis; and (iii) miR‐130, implicated in the inhibition of androgen receptor expression (Hu et al. 2017; Li et al. 2018; Yang et al. 2019; Xiao et al. 2024). In this context, a putative molecular control for miRNA functions in SCs may be exerted by circular RNAs (circRNAs), covalently closed RNA molecules produced by a back‐splicing mechanism (Memczak et al. 2013; Starke et al. 2015). Indeed, circRNAs, following their molecular biogenesis finely orchestrated by specific RNA‐binding proteins (RBPs), including fused protein in sarcoma (FUS), RNA polymerase II (RNApol2) and quaking (QKI), regulate gene expression at posttranscriptional level by a miRNA tethering activity in order to protect the related mRNA targets from degradation (Conn et al. 2015; Errichelli et al. 2017; Chioccarelli, Falco, et al. 2021). Recent investigations have revealed the potential implications of circRNAs in SC physiology, as an analysis of circRNA expression profiles in the testes of Sertoli cell‐only syndrome (SCOS) patients identified aberrantly expressed circRNAs involved in SC dysfunction (Zhu et al. 2021). However, no systematic study has yet highlighted circRNA‐dependent regulatory mechanisms controlling age‐related SC dysfunctions or identified putative circRNAs driving a competitive endogenous RNA (ceRNA) network (ceRNET) in the modulation of age‐dependent effects in SCs.

In the present study, we have characterized testicular and spermatic phenotype of aged C57BL/6J mice used as experimental models of physiological aging, with a focus on age‐related SC senescence mechanism. Specifically, we identified a set of spermatic circRNAs representative of age‐dependent poor‐quality SPZ. Among these, we focused on circAbcb9, built its ceRNET potentially involved in SC senescence, validated, and confirmed in vitro its role in SC senescence by siRNA and mimic‐miRNA experiments carried out in immortalized SC line TM4. Interestingly, the expression of circAbcb9 was also confirmed in human SPZ, suggesting a conserved role in mammals.

Finally, we highlighted, for the first time, a Sertolian FUS‐dependent circRNA biogenesis potentially involved in SC survival and homeostasis.

## 2 Results

### 2.1 Aging Process Impairs Spermatogenesis and Sperm Quality Parameters

**FIGURE 1:**
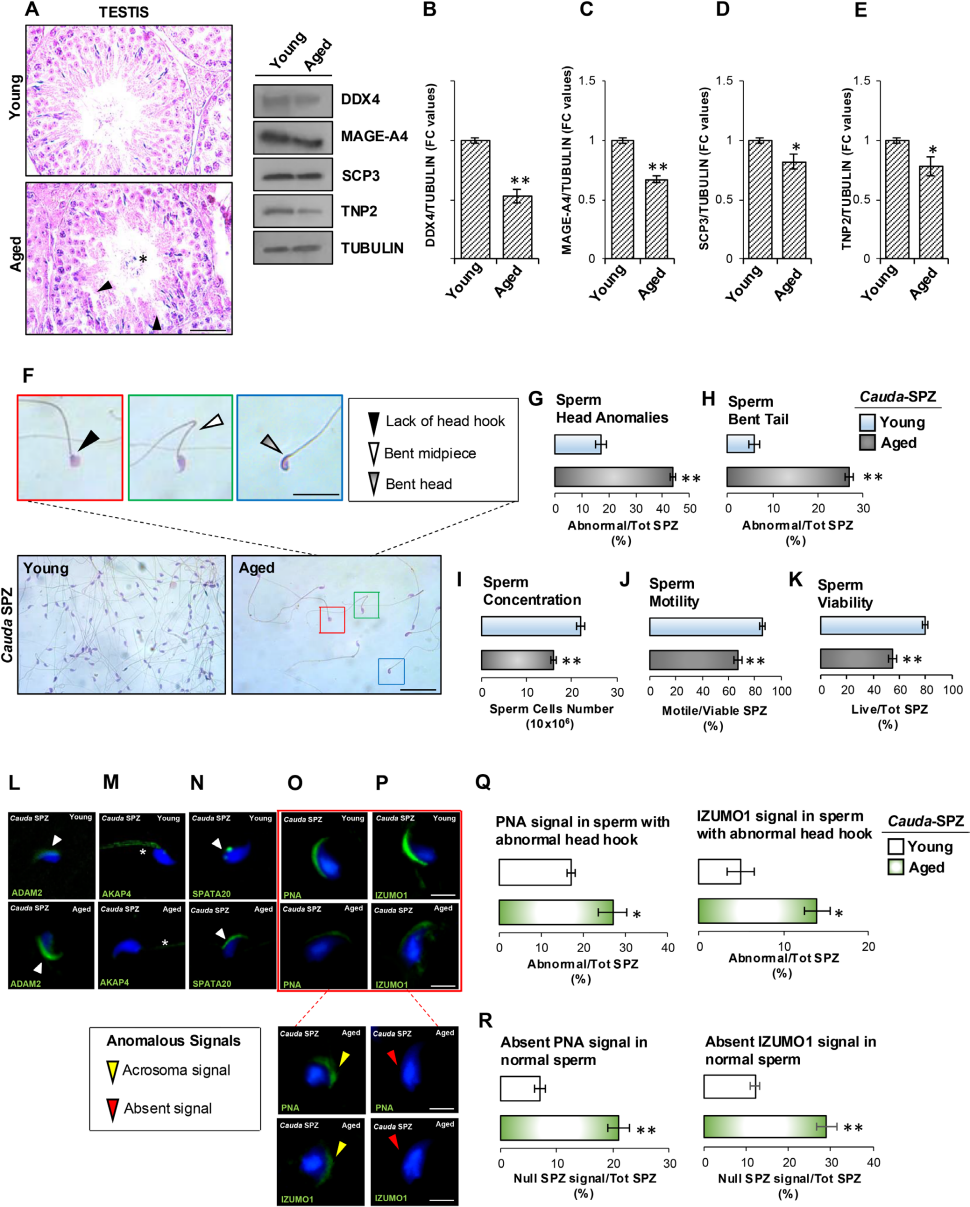
Testicular and spermatic phenotype of Aged mice. (A) H&E staining of Young (*n* = 6) and Aged (*n* = 6) testicular cross‐sections. Disorganized seminiferous epithelium and germ cell lumen infiltration were indicated by black arrowheads and asterisks, respectively; scale bar: 50 μm. (B–E) Western blot analysis of DDX4 (B), MAGE‐A4 (C), SCP3 (D), and TNP2 (E) in Young and Aged testis (*n* = 6 different samples in triplicate). Signals were quantified by densitometry analysis and normalized against TUBULIN. Data were expressed in fold change (FC) OD values and reported as mean ± SEM; **p < 0.01; *p < 0.05. (F) H&E staining of Young (*n* = 6) and Aged (*n* = 6) *cauda* SPZ; scale bar: 100 μm. Anomalous sperm head and tail were indicated in the inset; scale bar inset: 10 μm. (G–H) Percentage of anomalous sperm heads (G) and bent tail (H) in Young (*n* = 6) and Aged (*n* = 6) *cauda* SPZ; data were expressed as the percentage of abnormal sperm heads/total SPZ and abnormal sperm tails/total SPZ and reported as mean ± SEM; **p < 0.01. (I–K) Sperm count (I), sperm motility (J) and sperm viability (K) assay in Young and Aged *cauda* SPZ (*n* = 6 different samples in triplicate); data were expressed as total sperm cell number, percentage of motile/live SPZ and live/total SPZ, respectively, and reported as mean ± SEM; **p < 0.01. (L–P) Immunofluorescence analysis of ADAM2 (L), AKAP4 (M), SPATA20 (N), PNA (O) and IZUMO1 (P) (FITC—green) in Young (*n* = 6) and Aged (*n* = 6) *cauda* SPZ, Nuclei were labeled with DAPI (blue); scale bar: 5 μm. (Q–R) PNA (Q) and IZUMO1 (R) positive sperm cell count in Young and Aged *cauda* SPZ related to SPZ with abnormal head hook (Q) and with normal morphology (R) (*n* = 6 different samples in triplicate). Data were expressed as percentage of positive cells with abnormal head hook on total SPZ and reported as mean ± SEM; **p < 0.01.

Morphological analysis of testis, as well as morpho‐functional analyses of *cauda* SPZ collected from Young (2–4 months of age) and Aged (22–24 months of age) mice, were performed to investigate the aging‐related effects on male reproductive skills and to assess the validity of the experimental model. The H&E staining carried out on testicular cross‐sections showed a disorganized seminiferous epithelium in Aged testis, characterized by an affected columnar stratification of germ cells within the seminiferous epithelium associated with their detachment and consequent lumen infiltration (Figure 1A).

Western blot analysis of specific germ cell markers was performed to investigate age‐related effects on germ cell progression. These analyses revealed a significant reduction (*p* < 0.01; *p* < 0.05) of all germ cell markers, DDX4 (germ cell hallmark), MAGE‐A4 (spermatogonia), SCP3 (spermatocytes) and TNP2 (spermatids) in Aged compared to Young testis, confirming an impaired spermatogenesis age dependent (Figure 1B–E).

The H&E staining of *cauda* SPZ collected from Young and Aged mice showed a large number of Aged SPZ with structural defects in terms of lack of normal sperm head hook and appearance of bents at both sperm head and midpiece level (Figure 1F). In detail, a significant increase in the number of SPZ with head anomalies (*p* < 0.01) and bent tails (*p* < 0.01) was observed in the Aged versus Young experimental group (Figure 1G,H), confirming that affected spermatogenesis leads to the production of poor‐quality SPZ. According to previous data, the analysis of sperm functional parameters revealed a significant reduction in sperm count (*p* < 0.01), as well as sperm motility (*p* < 0.01) and viability (*p* < 0.01) in Aged compared to Young SPZ (Figure 1I–K).

In order to molecularly define the abnormal sperm phenotype observed in Aged mice, immunofluorescence analyses for selective sperm quality markers were performed in Aged versus Young SPZ. As reported, in *cauda* SPZ collected from Aged mice, ADAM2, a protein involved in sperm migration along the female reproductive tract, appeared localized to the acrosomal region similar to the staining pattern observed for Young SPZ but with a stronger labeling (Figure 1L), whereas a drastic reduction of signal of AKAP4, a structural component of the sperm fibrous sheath, was observed in the tail of Aged sperm (Figure 1M). Interestingly, SPATA20 signal appeared normally localized at the posterior part of the sperm head at the head‐tail coupling apparatus level in the Young experimental group, whereas a massive signal dislocation, confined to the acrosomal region, was observed in Aged SPZ (Figure 1N). Then, the acrosome morphology was evaluated by PNA immunofluorescence staining. As shown, in SPZ collected from Young mice, the PNA signal was confined to the half‐anterior part of the sperm head consisting of the acrosomal region (Figure 1O). In the Aged experimental group, we observed a severe PNA‐labeling reduction or absence in SPZ having a normal head shape. Surprisingly, a well‐defined PNA signal confined to the acrosomal region occurred in Aged SPZ with morphological head defects (Figure 1O). IZUMO1 labeling showed the same PNA localization trend characterized in Aged SPZ (Figure 1P). Relatively to the sperm population having an abnormal head hook, the sperm count using the acrosomal PNA and IZUMO1 localization as the inclusive analysis parameter showed a significant increase (*p* < 0.01) in the percentage of sperm cells with a positive PNA and IZUMO1 signal in Aged compared with Young SPZ (Figure 1Q). In addition, relative to the sperm population having normal head morphology, a significant increase (*p* < 0.01) in the percentage of sperm cells with null PNA and IZUMO1 labeling was observed in Aged SPZ (Figure 1R).

### 2.2 Aging‐Dependent Effects on Spermatic circRNAs and SC Survival

Considering the wide range of defects observed in Aged SPZ and the defective germ cell progression likely dependent on SC dysfunctions, which typically occur in the aged testis (Huang et al. 2023; Xiao et al. 2024), here we pointed to identify spermatic DE‐circRNAs responsive to testicular aging.

**FIGURE 2:**
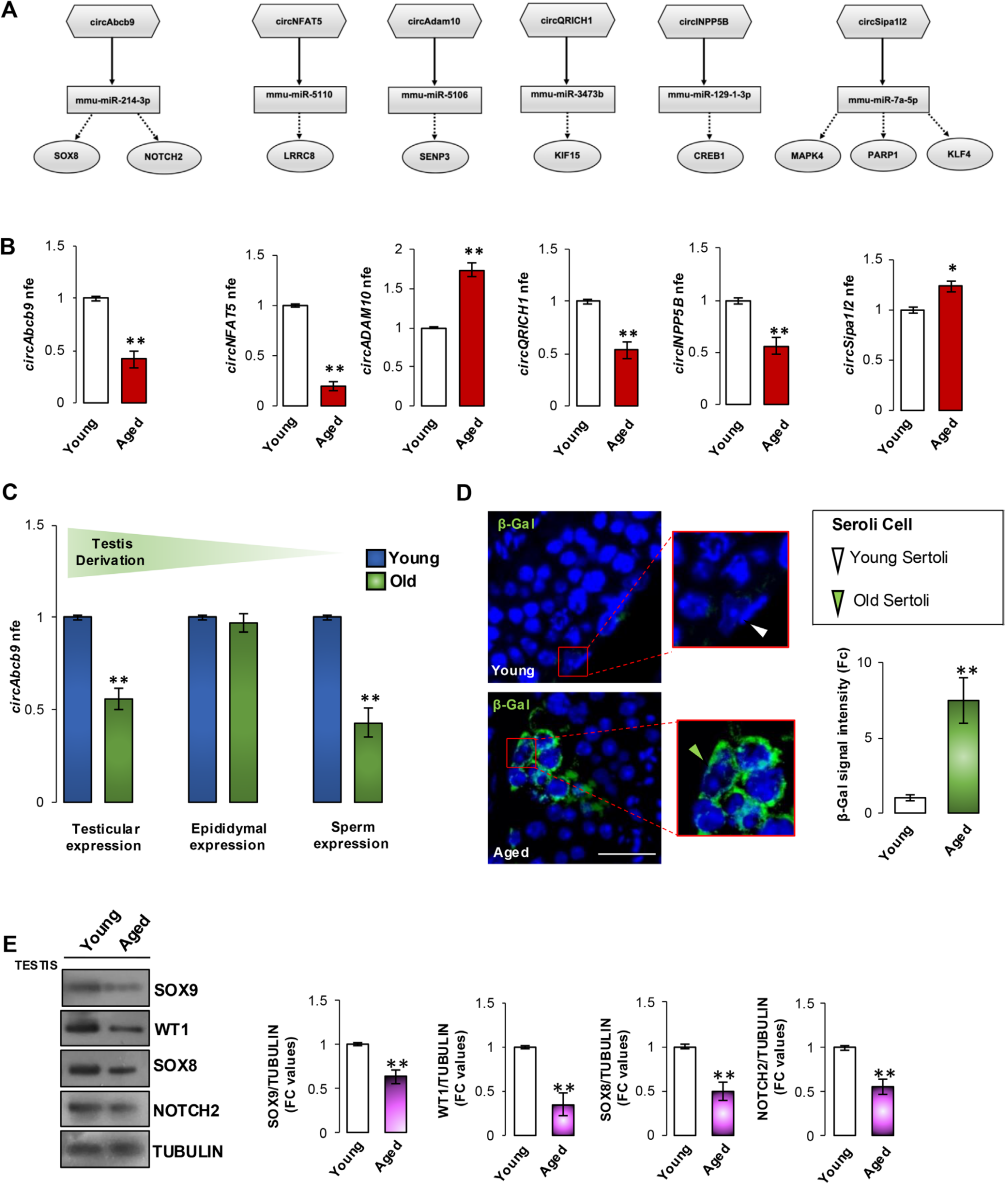
Aging‐dependent effects on sperm circRNA content and SC survival. (A) A set of spermatic circRNAs tethering a group of miRNAs and mRNAs as targets, all involved in SC dynamics. Networks were built using Cytoscape. Hexagonal and rectangular symbols represent circRNAs and miRNAs, respectively. The arrow indicates the tethering activity of circRNAs toward miRNAs, while the dotted arrow indicates the pathways downstream of the miRNAs. (B–C) Expression analysis of (B) 6 circRNAs in Young and Aged *cauda* SPZ (*n* = 6 in triplicate) and (C) circAbcb9 in testis, epididymis and *cauda* SPZ collected from Young and Aged mice (*n* = 6 in triplicate). RT‐qPCR data were normalized using *Cyclophilin*, expressed as fold expression (nfe), and reported as mean value ± S.E.M; **p < 0.01; *p < 0.05. (D) Senescence β‐gal immunofluorescence staining (green) of testicular tissues from Young and Aged mice (*n* = 6 independent experiments); scale bar: 20 μm. Representative images of SC staining were shown on the right. Results were expressed as fold change (Fc) relative to the Young group and reported as mean ± SEM. **p < 0.01. Nuclei were labeled with DAPI (blue). (E) Western blot analysis of SOX9, WT1, SOX8, and NOTCH2 in testis of Young and Aged mice (*n* = 6 in triplicate). Signals were quantified by densitometry analysis and normalized against TUBULIN. Data were expressed in fold change (FC) OD values and reported as mean ± SEM; **p < 0.01.

In particular, we chose an experimental approach aimed at identifying circRNAs applicable as markers of poor sperm quality due to SC decline age dependent. To achieve our goal, by using a bioinformatic approach, we built ceRNETs for six DE circRNAs (circAbcb9; circNFAT5; circAdam10; circQRICH1; circINPP5B; and circSipa1l2), extrapolated from our datasets and potentially responsive to aging, in order to identify the mRNA targets involved in SC dynamics. Bioinformatic analyses provided the top miRNA targets sponged by circRNAs (mmu‐miR‐214‐3p; mmu‐miR‐5110; mmu‐miR‐5106; mmu‐miR‐3473b; mmu‐miR‐129‐1‐3p; mmu‐miR‐7a‐5p) implicated in the regulation of SC‐related mRNAs such as: (i) KLFA, involved in SC differentiation (Godmann et al. 2008); (ii) SENP3, able to participate in pathways governing Sertolian blood–testis barrier dynamics (Wu et al. 2017); and (iii) KIF15, actively modulating changes in the organization of SC cytoskeleton (Wu et al. 2021) (Figure 2A).

First of all, we investigated the expression levels of the selected circRNAs in Young and Aged SPZ by RT‐qPCR analysis. Relatively to Young experimental group, all circRNAs analyzed appeared deregulated in Aged SPZ; in detail, as shown in Figure 2B, circAbcb9, circNFAT5, circQRICH1, and circINPP5B resulted significantly downregulated (*p* < 0.01; *p* < 0.05) while circAdam10 and circSipa1l2 appeared upregulated (*p* < 0.01) in Aged SPZ, suggesting an affected spermatic circRNA signature “prognostic” for SC aging.

To assess this hypothesis, we focused on the circAbcb9‐dependent network since its linear counterpart encoded for an ATP binding cassette protein able to interact with the lysosomal markers LAMP1 and LAMP2 and, more interestingly, is highly expressed in SCs, well known to exert prominent phagocytosis and secretory activities (Zhang et al. 2000). In agreement, both mRNAs, targets of circAbcb9‐ceRNET, are mainly involved in SC survivor pathways as follows: (i) SOX8 plays key roles in adult Sertoli cell function; indeed, its elimination results in progressive male infertility (Kennedy et al. 2007), and (ii) NOTCH2 has been recently reported as a target of the WT1 transcription factor, a key actor orchestrating SC homeostasis (Huang et al. 2023).

To deepen the downregulation of circAbcb9 observed in Aged SPZ, we evaluated its expression levels in SPZ, as well as in the testis and epididymis collected from Young and Aged mice. Relatively to Young experimental group, RT‐qPCR analysis showed a significant reduction (*p* < 0.01) of circAbcb9 levels in Aged testis and SPZ, while no differences were observed in the epididymal tissue (Figure 2C), suggesting a testicular origin for spermatic circAbcb9 deficiency. In order to understand if the testicular reduction of circAbcb9 could be associated with SC senescence, β‐gal staining was performed in Young and Aged testicular cross‐sections since β‐gal is an useful biomarker of cellular senescence both in vitro and in vivo (Debacq‐Chainiaux et al. 2009). As reported, a significant increase (*p* < 0.01) of β‐gal signal was observed in Aged testis, mainly at SC cytoplasm level (Figure 2D), suggesting an affected SC survival aging dependent. Consistently, western blot analysis revealed a significant testicular downregulation (*p* < 0.01) of SOX9, a known Sertoli cell marker (Hemendingerk et al. 2002), and WT1, a transcription factor needful for SC maintenance, as well as of circAbcb9‐ceRNET targets SOX8 and NOTCH2 (Figure 2E), suggesting the involvement of circAbcb9 in the aging‐related SC senescence.

### 2.3 CircAbcb9 in Human SPZ

In order to confirm a potential conserved role of circAbcb9 in age‐dependent fertility decline, we evaluated circAbcb9 expression in human SPZ. Firstly, we carried out bioinformatic analyses to identify the human circAbcb9 isoform having the most sequence homology to the murine counterpart. To achieve this aim, we used the CircAtlas 3.0 database (http://circatlas.biols.ac.cn/), suitable for mining in‐depth information about the candidate circRNA. Once all human circAbcb9 isoforms were identified, we individually aligned each human sequence with the murine one by using the Basic Local Alignment Search Tool (BLAST) (https://blast.ncbi.nlm.nih.gov/Blast.cgi). Among them, we found a hsa‐circAbcb9 isoform with a 98% homology relative to the murine circAbcb9 sequence. In addition, miRanda bioinformatic analysis included in CircAtlas 3.0 database showed that the selected hsa‐circAbcb9 isoform possessed the binding sites for the hsa‐miR‐214‐3p, confirming that the sponge activity of circAbcb9 toward miR‐214‐3p was conserved in both mammalian species. Reasoning that, we built the human circAbcb9‐ceRNET to identify the mRNA targets potentially involved in SC dynamics, similarly to the murine model (Figure S S1A). Interestingly, the bioinformatic analysis confirmed the key role of the circAbcb9/hsa‐miR‐214‐3p axis in the regulation of SC‐related mRNAs such as: (i) CLDN5, a tight junction protein modulating Sertolian blood–testis barrier functions (Morrow et al. 2009); (ii) SMAD3 that participates in the modulation of signaling pathways driving Sertoli cell proliferation and maturation (Itman et al. 2011); and (iii) WNT3, a morphogen of the Wnt/β‐catenin pathway implicated in Sertoli cell‐mediated regulation of spermatogenesis, crucial for male fertility (Basu et al. 2018). The miR‐214‐3p targets SOX8 and NOTCH2 were also preserved in the human circAbcb9‐ceRNET, reinforcing the conceivable conserved role for circAbcb9 as a spermatic epigenetic marker related to age‐dependent fertility decline.

To confirm this aspect, we investigated the expression levels of the identified hsa‐circAbcb9 in human SPZ by RT‐qPCR analysis. In detail, we aimed to assess spermatic hsa‐circAbcb9 content in (i) high‐ (A‐SPZ) and low‐ (B‐SPZ) quality spermatozoa fractions collected from normozoospermic men, in order to correlate the expression of circAbcb9 to sperm morpho‐functional parameters (Chioccarelli et al. 2019), and (ii) A‐SPZ fraction collected from Young and Aged men, in order to assess circAbcb9 content in sperm fractions having the greatest fertilizing abilities in relation to aging. As shown in Figure S1B, the levels of hsa‐circAbcb9 were significantly downregulated (*p* < 0.01) in B‐SPZ, suggesting an affected spermatic circAbcb9 content relative to poor‐quality sperm parameters. Accordingly, a significant reduction (*p* < 0.01) of hsa‐circAbcb9 was observed in Aged as compared to Young A‐SPZ (Figure S1C), confirming the putative role of circAbcb9 as a prognostic marker for age‐dependent fertility decline.

### 2.4 CircAbcb9‐ceRNET is Involved in SC Senescence

**FIGURE 3:**
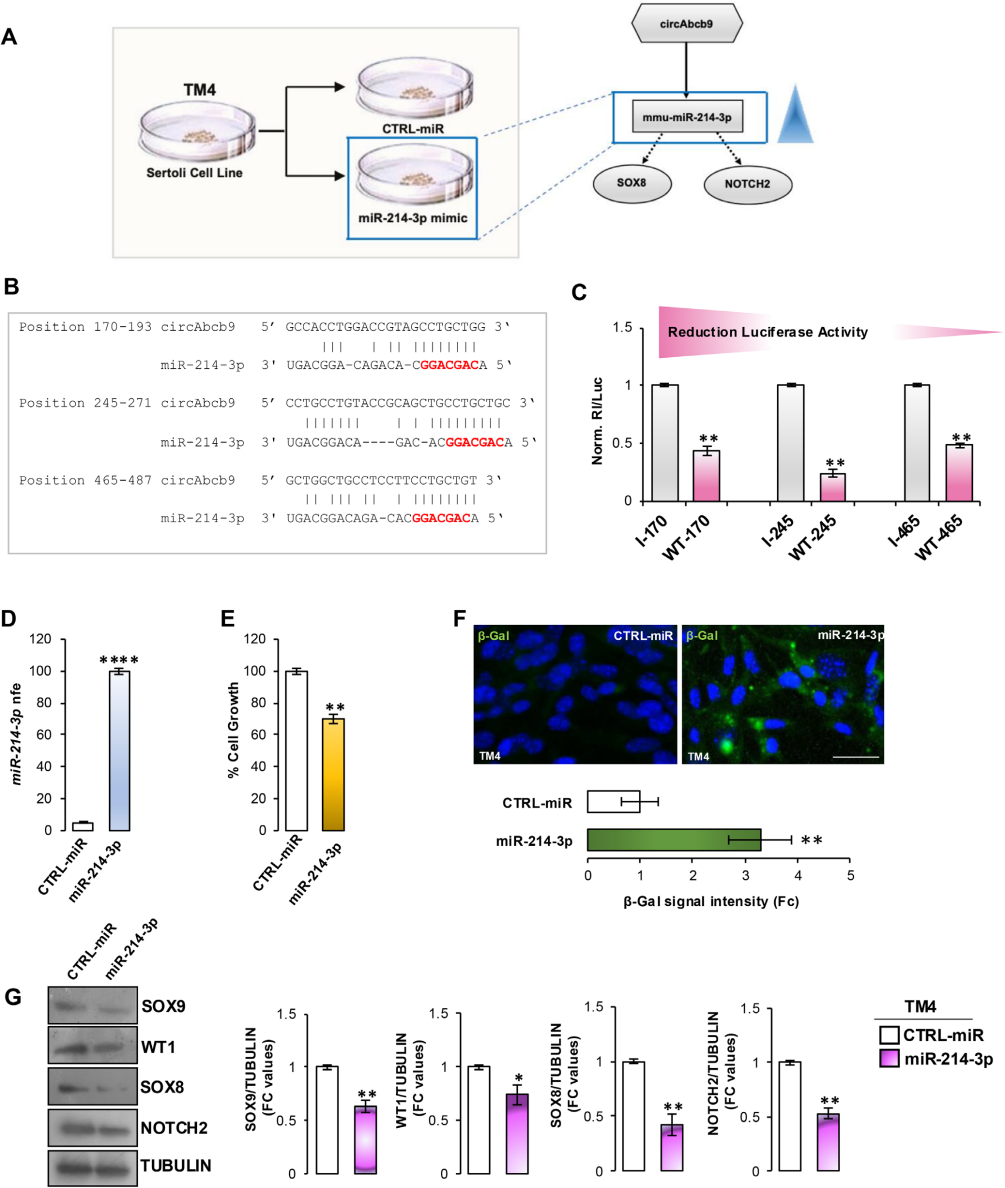
miR‐214‐3p modulates SC senescence. (A) A schematic representation of the in vitro experimental strategy. (B) Prediction of miR‐214‐3p binding sites on circAbcb9 sequence (miRNA seed sequence is indicated in red). (C) TM4 cells transfection with the luciferase‐based reporter constructs containing the wild‐type target sequence of miR‐214‐3p (WT—starting position number of the target pairing) or the control plasmids with inverted target sequence (I—starting position number of the target pairing) along with 50 nM of the miRNA mimic (*n* = 6 independent experiments). The Renilla luciferase activity (Rl) was recorded and normalized to the firefly luciferase activity (Luc); values were reported as fold mean ± S.E.M. relative to RL/Luc recorded for transfection of controls (specific I construct + miRNA mimic) which were set to 1; **p < 0.01. (D) Expression analysis of miR‐214‐3p in TM4 cells following transfection with an unrelated control (CTRL‐miR) or miR‐214‐3p mimic at 50 nM; (*n* = 6 independent experiments); RT‐qPCR data were normalized using *U6 snRNA*, expressed as fold expression (nfe) and reported as mean value ± S.E.M; ****p < 0.0001. (E) Cell growth analysis in TM4 cells following miR‐214‐3p mimic transfection by using MTT assay (*n* = 6 independent experiments). Data were normalized against to CTRL group, expressed as percentage of cell growth, and reported as mean ± SEM; **p < 0.01; (F) Senescence β‐gal immunofluorescence staining (green) in TM4 cells following miR‐214‐3p mimic transfection (*n* = 6 independent experiments). Data were expressed as fold change (Fc) relative to the CTRL group and reported as mean ± SEM. **p < 0.01. Nuclei were labeled with DAPI (blue); scale bar: 50 μm. (G) Western blot analysis of SOX9, WT1, SOX8, and NOTCH2 proteins in TM4 cells following miR‐214‐3p mimic transfection (*n* = 6 in triplicate). Signals were quantified by densitometry analysis and normalized against to TUBULIN. Data were expressed in fold change (FC) OD values and reported as mean ± SEM; **p < 0.01.

To confirm the regulatory role of circAbcb9‐ceRNET in SC senescence, we set up an in vitro experimental strategy to selectively investigate SC responsiveness to circAbcb9‐mediated molecular mechanisms. In detail, we pointed to upregulate miR‐214‐3p expression in the immortalized SC line TM4 in order to affect mRNA targets and, in turn, trigger cellular senescence downstream (Figure 3A). Reasoning that, we firstly assessed the expression of circAbcb9 network components in TM4 cells; both circAbcb9 and miR‐214‐3p were expressed in the immortalized SC line (data not shown). A bioinformatic approach revealed three miR‐214‐3p predicted binding sites on circAbcb9 (Figure 3B). In order to demonstrate that miR‐214‐3p was able to directly bind to those circAbcb9 sequences, we carried out an experimental validation based on luciferase reporter constructs transfected into the TM4 cell line. Thus, circAbcb9 sequences predicted as miR‐214‐3p target sites (Sequence 170–193, Sequence 245–271, and Sequence 465–487) were singularly cloned downstream of the *Renilla reniformis* luciferase (Rl) coding sequence carried by the psi‐Check‐2 vector. Then, the obtained reporter constructs were transfected into TM4 cells along with miR‐214‐3p mimics, and luciferase activity was evaluated. As shown, all circAbcb9 cloned sequences significantly (*p* < 0.01) reduced luciferase activity compared to the control reporter vectors (indicated as I) (Figure 3C), confirming that miR‐214‐3p was able to directly bind to circAbcb9. After that, the miR‐214‐3p mimic was transfected into TM4 cells in order to evaluate the hypothesized potential promotion of SC senescence mediated by the relative mRNA target downregulation. Following transfection, a significant increase (*p* < 0.01) of miR‐214‐3p levels was observed (Figure 3D), confirming the success of the experimental procedure. The overexpression of miR‐214‐3p caused a cell growth approximately 30% lower (*p* < 0.01) than the control group (Figure 3E), phenotypically associated with a prominent TM4 cellular senescence, highlighted by the significant increase (*p* < 0.01) of β‐gal signal (Figure 3F). Consistently, the Western blot analysis showed a significant reduction (*p* < 0.01; *p* < 0.05) of SC markers SOX9 and WT1 and especially of miR‐214‐3p targets SOX8 and NOTCH2 (Figure 3G), demonstrating the regulatory role of the circAbcb9 network in the modulation of SC senescence.

### 2.5 FUS‐Driven circAbcb9 Biogenesis Modulated SC Senescence

**FIGURE 4:**
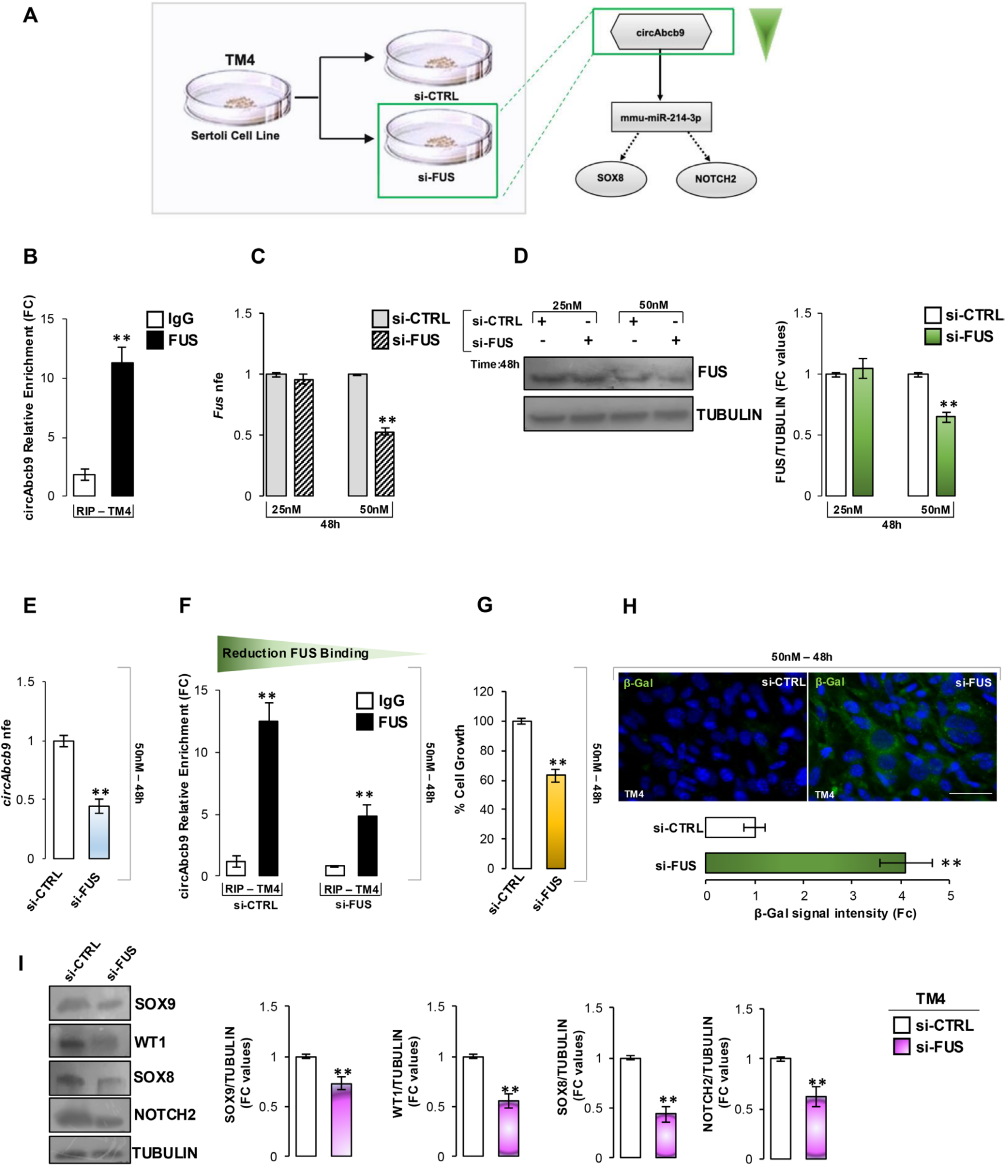
FUS protein regulates sertolian circAbcb9 biogenesis and in turn cellular senescence. (A) A schematic representation of the in vitro experimental strategy. (B and F). CircAbcb9 enrichment levels in the products of RIP assay (FUS‐IP vs IgG‐IP) in (B) TM4 cell line (*n* = 6 independent experiments) and (F) TM4 cells treated with siCTRL or siFUS (50 nM) (*n* = 6 in triplicate) by RT‐qPCR. Data were expressed in fold change (FC) and reported as mean ± SEM; **p < 0.01. (C‐D) Expression analysis of (C) *Fus* transcript and (D) FUS protein in TM4 cells treated with siCTRL or siFUS (25 and 50 nM) (*n* = 6 in triplicate); RT‐qPCR data were normalized using *Cyclophilin*, expressed as fold expression (nfe), Western blot signals were quantified by densitometry analysis, normalized against to TUBULIN, expressed in fold change (FC) OD values and reported as mean ± SEM; **p < 0.01. (E) CircAbcb9 levels in TM4 cells treated with siCTRL or siFUS (50 nM) (*n* = 6 in triplicate). Data were normalized using *Cyclophilin*, expressed as nfe, and reported as mean ± S.E.M; **p < 0.01. (G) Cell growth analysis in TM4 cells using MTT assay following transfection with siCTRL or siFUS (50 nM) (*n* = 6 independent experiments). Data were normalized against to siCTRL group, expressed as percentage of cell growth, and reported as mean ± SEM; **p < 0.01; (H) Senescence β‐gal immunofluorescence staining (green) in TM4 cells treated with siCTRL or siFUS (50 nM) (*n* = 6 independent experiments). Data were expressed as fold change (Fc) relative to the siCTRL group, and reported as mean ± SEM. **p < 0.01; scale bar: 50 μm. (I) Western blot analysis of SOX9, WT1, SOX8, and NOTCH2 in TM4 cells treated with siCTRL or siFUS (50 nM) (*n* = 6 in triplicate). Signals were quantified by densitometry analysis and normalized against TUBULIN. Data were expressed in fold change (FC) OD values and reported as mean ± SEM; **p < 0.01.

With the aim to corroborate the central role of circAbcb9 in the regulation of SC senescence, we set up a second in vitro experimental approach pointed to recapitulate circAbcb9 downregulation previously characterized in Aged testis. Thus, we affected the Sertolian back‐splicing mechanism by silencing the endogenous *Fus* expression through an RNA interference strategy (Figure 4A). First of all, FUS expression was verified in TM4 cells (data not shown). In addition, we carried out an RNA‐binding protein immunoprecipitation (RIP) assay by using FUS antibody to assess the physical interaction between FUS and circAbcb9 in TM4 cells. Relatively to the use of control IgG, the results showed a significant 11.28‐fold enrichment of circAbcb9 (*p* < 0.01) when the anti‐FUS Ab was used (Figure 4B), reinforcing the idea of a direct interaction between FUS protein and circAbcb9, and in turn, a potential FUS‐dependent back‐splicing machinery in SCs. A specific pool of siRNA for *Fus* mRNA (siFus) was transfected in TM4 cells at different doses (25 and 50 nM) for 48 h. The RT‐qPCR analysis showed a significant downregulation of *Fus* expression levels (*p* < 0.01) at the dose of 50 nM in comparison to its unrelated negative control molecule (siCTRL) (Figure 4C), also supported by the significant reduction of FUS protein (*p* < 0.01) observed at the same dose (Figure 4D). Based on these results, we carried out the downstream investigations choosing the 50 nM dose. As expected, a significant reduction of circAbcb9 levels (*p* < 0.01) was observed in TM4 cells transfected with siFUS (Figure 4E), suggesting the direct involvement of FUS protein in circAbcb9 biogenesis. In agreement, relative to the use of control IgG, the RIP assay by using FUS antibody revealed a significant 12.56‐ and 4.82‐fold enrichment of circAbcb9 (*p* < 0.01) in siCTRL and siFUS experimental groups, respectively (Figure 4F), confirming the affected circAbcb9 biogenesis following the in vitro *Fus* silencing. A significant downregulation of TM4 cell growth (*p* < 0.01) was also observed in the siFUS compared to the siCTRL group (Figure 4G), morphologically coupled to a significant increase (*p* < 0.01) of β‐gal signal (Figure 4H), confirming that circAbcb9 downregulation, dependent on *Fus* silencing, promoted a cellular senescence phenotype. The Western blot analysis showed a significant reduction (*p* < 0.01) of SOX9 and WT1 proteins in the siFUS compared to the siCTRL group, as well as of circAbcb9‐network targets SOX8 and NOTCH2 (Figure 4I) confirming the involvement of circAbcb9‐ceRNET in SC senescence.

## 3 Discussion

Spermatogenesis process ensures lifelong male reproductive capability although its functional aging‐dependent decline compromises the ability to successfully achieve a pregnancy and increases the risk of male reproductive diseases (Eskenazi et al. 2003; Morales et al. 2003; Kovac et al. 2013; Begueria et al. 2014). In recent years, circRNAs have emerged as important regulators of male fertility mainly acting as: (i) biological molecules participating in testicular development and spermatogenesis processes; (ii) biomarkers associated with male infertility conditions (Kyrgiafini and Zissis Mamuris 2023; Tang et al. 2023).

To date, a comprehensive study focused on the role of circRNAs in aging‐dependent reproductive disorders has been lacking. Therefore, we conducted a fine molecular investigation aimed at identifying circRNAs as part of functional ceRNETs related to testicular aging.

In aged mice, several reproductive anomalies were observed. Firstly, we found an impaired integrity of the seminiferous epithelium affecting spermatogenesis progression, as evidenced by the reduction of testicular germ cell subpopulations. In detail, DDX4 protein was used as a germ cell hallmark while MAGE‐A4, SCP3, and TNP2 proteins were used to selectively investigate the content of spermatogonia, spermatocytes, and spermatids, respectively (Chioccarelli, Migliaccio, et al. 2021). The decrease of MAGE‐A4 spermatogonia‐related molecular marker, as well as of SCP3 and TNP2, markers for spermatocytes and spermatids, respectively, correlated with the decreased SPZ number occurring in aged mice. Consistent with our results, previous studies have already reported impaired SPZ production due to the decreased proliferation of undifferentiated spermatogonia and spermatocyte apoptosis (Morales et al. 2003; Kokkinaki et al. 2010; Vrooman et al. 2014; Pohl et al. 2019; Endo et al. 2024), thus supporting the validity of our murine experimental aging model.

As expected, Aged SPZ were characterized by a wide set of abnormal morpho‐functional features, beyond number and motility. They exhibit structural defects such as bent head or bent midpiece as well as a lack of normal head hook. Similar spermatic phenotypes have been reported by Endo and coauthors (Endo et al. 2024), but here we have deeply investigated this aspect, analyzing structural sperm markers such as (i) ADAM2 protein, actively involved in sperm migration along the female reproductive tract (Xiong et al. 2019); (ii) AKAP4, a scaffold protein required for the integrity of the fibrous sheath and sperm motility (Miki et al. 2022); (iii) SPATA20 protein, responsible for the correct assembly of head‐tail coupling apparatus (Wang et al. 2023); and (iv) peanut lectin PNA and IZUMO1 membrane glycoprotein to assess the acrosome state and sperm–oocyte interaction skills, respectively (Martinez et al. 2023).

Consistent with the reduction of flagellar AKAP4 signal observed in Aged SPZ, male mice lacking the AKAP4 protein failed to properly organize the fibrous sheath compromising sperm motility (Miki et al., 2002). Likewise, SPATA20 mislocation of head–tail coupling apparatus may be responsible for bent head onset in Aged SPZ, given that nonsense mutations driving SPATA20 loss‐of‐function have been associated with defects in sperm head–tail conjunction (Wang et al. 2023).

Interestingly, PNA staining used for the acrosome state evaluation appeared to vary depending on the sperm head shape type. SPZ with normal head shapes exhibited a loss or reduction of PNA signal, while deformed SPZ maintained normal PNA staining. In line with this observation, a similar trend was characterized for the IZUMO1 glycoprotein, almost suggesting enhanced fertilization capabilities in morphologically defective SPZ.

We have previously profiled differentially expressed circRNAs (DE‐circRNAs) in SPZ collected from mice harboring physio‐pathological conditions (Manfrevola et al. 2022, 2023), highlighting a tight correlation between sperm morpho‐functional anomalies and spermatic circRNA cargo. Thus, a fascinating aspect explored in this study consists of how much the aging process may affect spermatic circRNA cargo, given the emerging role of these epigenetic molecules as key regulators of male reproduction and sperm fertilization potential (Chioccarelli, Falco, et al. 2021; Manfrevola et al. 2022, 2023; Mele et al. 2024). In this study, we identified circAbcb9 as a biomarker of aging‐related spermatic dysfunctions and a master regulator of SC senescence at the testicular level. The experimental validation of circAbcb9 demonstrated a prominent downregulation in both Aged testis and SPZ, strengthening the idea that anomalous circAbcb9 regulation originates in the testis and persists in sperm cells as a marker of testicular age‐related senescence. In agreement, the similar responsiveness of circAbcb9 content to the aging process in both murine and human SPZ strongly highlights a conceivable role for circAbcb9 as a prognostic marker of age‐dependent fertility decline conserved across mammals.

In support of this hypothesis, the construction of circAbcb9‐ceRNET allowed us to identify the predicted mRNA targets all involved in the regulation of SC functions. It is not a coincidence that bioinformatic analyses revealed Sertolian targets as essential for spermatogenesis maintenance as they provide structural support and nutrients to germ cells. However, they also appear highly vulnerable to the effects of aging, being the main cell type to suffer from age‐related decline (Petersen and Soder 2006; Nie et al. 2022; Huang et al. 2023; Xiao et al. 2024).

Age‐related reduction in SC content, as highlighted by SOX9 downregulation and the increase of cellular senescence, further supports our hypothesis, thus making the circAbcb9/miR‐214‐3pmRNA targets axis closely related to SC homeostasis. Further confirmations for this came from the observed reduction of SOX8 and NOTCH2 proteins, predicted targets of circAbcb9‐ceRNET. Consistently, *Sox8* knockout males show Sertoli cell dysfunctions leading to spermatogenic cycle dysregulation (Kennedy et al. 2007). Remarkably, recent findings have reported a perturbed aging‐dependent transcriptional profile in SCs identifying the WT1 transcription factor essential for SC homeostasis since its age‐dependent downregulation closely correlates with cellular senescence onset (Huang et al. 2023). The reduction of the WT1 factor and of its related target NOTCH2 in Aged testis strongly led us to persevere in circAbcb9‐network investigations.

With this in mind, we set two independent in vitro experimental approaches in immortalized SC line‐TM4 in order to confirm the involvement of circAbcb9‐ceRNET in cellular senescence. First, we validated the ability of circAbcb9 to directly bind to miR‐214‐3p on three different sequences using a Luciferase reporter assay strategy. We then transfected miR‐214‐3p mimic in TM4 cells to molecularly demonstrate the correlation between miRNA targets downregulation and cellular senescence. As expected, the upregulation of miR‐214‐3p promoted SC senescence associated with reductions of SOX8 and NOTCH2 miRNA targets as well as Sertolian markers SOX9 and WT1.

Next, to definitively confirm the key role of circAbcb9 in SC senescence, we downregulate the endogenous circAbcb9 content in TM4 cells, aiming to in vitro recapitulate the same age‐related phenotype characterized in vivo. To achieve our goal, we chose to investigate the possible role of FUS protein, the main RBP involved in the back‐splicing mechanism (Chioccarelli, Falco, et al. 2021), in the modulation of Sertolian circAbcb9 biogenesis. Thus, we silenced the endogenous *Fus* expression by RNA interference experiments and revealed: (i) downregulation of circAbc9 content; (ii) reduced physical interaction between FUS and circAbcb9; and (iii) induction of cellular senescence molecularly correlated with the downregulation of circAbcb9‐ceRNET‐related targets, thus corroborating the involvement of circAbcb9‐ceRNET in SC senescence.

Collectively, our study provides a new fascinating role for circRNAs in modulating SC homeostasis in the context of aging.

## 4 Conclusions

We present a comprehensive functional study aimed at deepening our understanding of the molecular mechanisms underlying testicular aging. Our findings demonstrate the key role of circAbcb9‐ceRNET in regulating age‐related SC senescence. Furthermore, our work provides new outcomes regarding spermatic circRNAs as potential biomarkers suitable for sperm quality evaluation and earlier clinical diagnosis of age‐dependent fertility decline. Finally, we report for the first time a FUS‐dependent back‐splicing activity in SCs, crucial for maintaining SC homeostasis.

## 5 Limitations and Future Perspectives

Our study provides new findings regarding the role of circAbcb9‐ceRNET in regulating age‐related SC senescence. Here, we deliberately investigated and validated circAbcb9‐ceRNET in SCs since the relative molecular targets identified by a bioinformatic approach appeared mainly involved in the regulation of SC functions. In addition, a potential role of circAbcb9 as a spermatic molecular marker prognostic for age‐dependent fertility decline is demonstrated in both murine and human SPZ, strongly highlighting a translational potential conserved across mammalian species.

However, this study shows some limitations that need to be acknowledged. The validation of circAbcb9‐ceRNET should be performed in cultured human‐derived SCs as well as in alternative experimental models such as conditional knockout mice. Future experimental approaches will be planned to elucidate these aspects. Despite these challenges, our study provides valuable preliminary insights into a new and unexplored role of circRNAs in the molecular networks underlying SC functions.

## 6 Material and Methods

### 6.1 Animals

Wild‐type C57BL/6J mice were purchased from (ENVIGO Srl, Udine, Italy). Experiments were performed on male mice at two age‐time points: 2–4 months of age was used as a time point ensuring young life and sexual maturation showing high fertilizing abilities (Young mice), while 22–24 months of age represents the temporal window related to the age‐dependent fertility decline and was used as the aged time point based on C57BL/6J average lifespan (around 29 months) and previous studies on aging (Aged mice) (Endo et al. 2024).

All procedures were carried out in accordance with the National Research Council's publication Guide for Care and Use of Laboratory Animals and approved by the Italian Ministry of Education and the Italian Ministry of Health, with authorization n°405/2021‐PR. The animals were maintained in a temperature‐controlled room at 22°C under a 12 h dark/light cycle and fed a pellet diet with free access to water. The animals were sacrificed by cervical dislocation following a full sedation with 4% isoflurane (Iso‐Vet, Piramal Healthcare, UK Limited) for 5 min in a Plexiglass chamber. The testes were rapidly removed and stored at –80°C for molecular investigations and/or fixed for morphological analyses. The epididymides were processed for the collection of SPZ from the *cauda* region (*cauda* SPZ), which represents the mature spermatic population with the highest fertilizing abilities. The number of animals needed for this study was established through the G*Power analysis (latest ver. 3.1.9.7; Heinrich‐Heine‐Universität Düsseldorf, Düsseldorf, Germany; http://www.gpower.hhu.de/) required to get the permission for in vivo experiments in Italy, suggested by the Legal Entity giving the permission.

### 6.2 Histological Analysis

Testes from Young (*n* = 6) and Aged (*n* = 6) mice were collected and fixed in Bouin's solution overnight (O.N.) to perform morphological investigations. Following the fixation step, the samples were dehydrated in ethanol, cleared in xylene, and embedded in paraffin using standard procedures. Testicular sections (7 μm thick) were deparaffined and stained with hematoxylin and eosin (H&E) reagents according to the standard protocol. Histological analyses were conducted under a light microscope (Leica CTR500, Leica Microsystems Inc., Milan, Italy) and images were captured using a high‐resolution digital camera (Leica DC300F). A minimum of 10 random sections was analyzed for each sample. All the results were validated twice by the same operator.

### 6.3 Mouse Sperm Collection

*Cauda* epididymides collected from different Young (*n* = 6) and Aged (*n* = 6) mice were separately cut in PBS (pH 7.6) to let SPZ flow out from the ducts and filtered throughout cheesecloth. Following a centrifugation at 1500 *g* for 30 min at 4°C, SPZ pellets were incubated on ice for 30 min with Somatic Cell Lysis Buffer (SCLB; 0.1% SDS, 0.5% Triton X‐100 in DEPC‐H2O) to eliminate somatic cell contaminations. After two washes in PBS, aliquots of *cauda* SPZ were stored at –80°C for molecular analyses and/or dried on slides to be stored at –20°C for morphological investigations. For sperm functional analysis, aliquots of *cauda* SPZ were processed as reported above.

### 6.4 Mouse Sperm Functional Analysis

Aliquots of *cauda* SPZ collected from Young (*n* = 6) and Aged (*n* = 6) mice were used to assess sperm functional parameters. The number of motile, live, and total SPZ was investigated using a hemocytometer (Burker Chamber) under a light microscope (Leica CTR500, Leica Microsystems Inc., Milan, Italy). For the total SPZ number, 20 fields per sample were analyzed (mean value ± SEM). The viable dye Trypan blue reagent (Trypan Blue, 0.4% Solution, 17‐942E Lonza) was used to evaluate the number of live SPZ, calculated as the percentage of live/total SPZ (mean value ± SEM). Motile SPZ were reported as a percentage of motile/live SPZ (mean value ± SEM). A minimum of 100 sperm cells was counted for each analysis (*n* = 6 for each experimental group in triplicate). All the results were validated twice by the same operator.

### 6.5 Mouse Sperm Morphological Analysis

Aliquots of *cauda* SPZ collected from Young (*n* = 6) and Aged (*n* = 6) mice were dried on slides and processed for H&E staining. Morphological analyses were performed under a light microscope (Leica CTR500, Leica Microsystems Inc., Milan, Italy) capturing images by using a high‐resolution digital camera (Leica DC300F). The number of SPZ with anomalous head and tail morphology was quantified by performing a cell counting assay (*n* = 6 for each experimental group in triplicate). A minimum of 100 sperm cells was counted for each assay, and data were reported as the percentage of anomalous sperm heads/total SPZ (mean value ± SEM). All the results were validated twice by the same operator.

### 6.6 Mouse Sperm Immunofluorescence Analysis

Aliquots of *cauda* SPZ collected from Young (*n* = 6) and Aged (*n* = 6) mice were dried on slides, fixed in 4% paraformaldehyde (sc‐281692; Santa Cruz Biotechnology, Heidelberg, Germany) for 20 min at room temperature, and permeabilized with 0.1% Triton X‐100 (X100; Sigma‐Aldrich, Milano, Italy). Following the blocking step with 10% donkey serum (ab7475; Abcam, Cambridge, UK), slides were incubated with different primary antibodies diluted 1:100 [IZUMO1 (ab211623; Abcam, Cambridge, UK), AKAP4 (HPA020046), ADAM2 (HPA026581), SPATA20 (HPA031442); Sigma‐Aldrich, Milano, Italy] O.N. at 4°C. A FITC‐conjugate antibody (711‐095‐152; Jackson ImmunoResearch, Cambridge, UK) was used as a secondary antibody (diluted 1:100) for 1 h at 37°C. To assess acrosome state, slides were stained with peanut agglutinin (PNA) lectin (L21409; Alexa Fluor 488, Thermo Fisher Scientific, Waltham, MA, USA), diluted 1:50, for 1 h at 37°C, following the permeabilization step carried out with 0.1% Triton X‐100 (X100; Sigma‐Aldrich, Milano, Italy). For nuclei labeling, DAPI (D9542; Sigma‐Aldrich, Milano, Italy) was used. Samples were analyzed under an optical microscope (Leica DM 5000 B + CTR 5000; Leica Microsystems, Wetzlar, Germany) with a UV lamp, and images were captured by using a Leica DFC320 R2 digital camera. To quantify the number of PNA and IZUMO1 positive cells, relative to SPZ with normal or abnormal head morphology, a minimum of 100 sperm cells was counted (*n* = 6 for each experimental group in triplicate) for each assay. Data were reported as the percentage of PNA and IZUMO1 positive cells in both sperm with abnormal head/total SPZ and sperm with normal head/total SPZ (mean value ± SEM). All the results were validated twice by the same operator.

### 6.7 Human Sperm Collection

Human semen samples were collected from (i) normozoospermic men (*n =* 6), and (ii) Young (range of age from 25 to 35 years) and Aged (range of age from 58 to 65 years) men (*n* = 6 for each experimental group). The samples were produced by masturbation following 5–7 days of sexual abstinence as previously reported (Mele et al. 2024). In accordance with the World Health Organization (2010) reference criteria, semen parameters were assessed by using a computer‐aided sperm analyzer (CASA), the Sperm Class Analyzer (SCA) system (SCA version 6.1; Microptic, S.L. Viladomat, Barcelona, Spain) in order to classify normozoospermic men. After liquefaction for 30 min at 37°C and semen parameters analysis, the high‐quality sperm fraction (A‐SPZ) was purified and separated from the low‐quality sperm fraction (B‐SPZ) using a density gradient centrifugation approach. In detail, semen was loaded at the top of a 40%/80% discontinuous PureCeption (Cooper Surgical, Trumbull, CT, USA) gradient and centrifuged at 300*g* for 20 min. Following centrifugation, the A‐SPZ fraction was purified from 80% PureCeption, while the B‐SPZ fraction was purified from 40% PureCeption. Both fractions were washed with sperm washing medium (HTF‐IrvineScientific) and treated with somatic cell lysis buffer (SCLB) (0.1% SDS, 0.5% Triton X‐100 in DEPC‐H_2_O) for 30 min on ice to eliminate any somatic cell contamination. Finally, SPZ from each experimental group were counted under a light microscope to pellet equal concentrations of sperm cells (1 × 10^7^ cells) and stored at –80°C for molecular investigations.

### 6.8 Reporter Constructs

The circAbcb9 sequences predicted to be targeted by miR‐214‐3p were chemically synthesized and cloned into the psiCheck‐2 vector (Promega, Madison, WI, USA). In brief, couples of oligonucleotides, representing the target sites (code WT) and carrying additional upstream XhoI and EcoRV restriction sites and a downstream NotI site, were annealed and ligated into the XhoI and NotI sites of the psiCheck‐2 vector; EcoRV digestions and sequencing were then used to identify recombinant clones. Control plasmids (code I) were obtained by the same approach, with the exception that the cloned couple of oligonucleotides represented the inverted target sequence.

### 6.9 Cell Culture, Transfections and Luciferase Assays

Murine SC line TM4 (Icellbioscience iCell‐m059) was cultured in DMEM/F12 (1:1) supplemented with 2.5% fetal bovine serum, 5% horse serum, 50 U/mL penicillin, and 100 mg/mL streptomycin. The day before transfection, the cells were trypsinized and seeded in a medium without antibiotics in 12‐well plates. Cells at 80%–90% of confluence were transfected by using 3 of Lipofectamine 2000 (Invitrogen) for 1 μg of nucleic acids with: (i) 50 nM miRIDIAN mmu‐miR‐214‐3p mimic or its unrelated sequence negative control miRNA mimic (Dharmacon–Horizon Discovery, Cambridge, UK) along with 0.2 μg of reporter constructs for the luciferase assays, or alone for cell proliferation and senescence assays (*n* = 6 independent experiments); and (ii) 50 nM MISSION siRNA against FUS (siFUS) or its unrelated MISSION siRNA negative control (siCTRL) (Sigma‐Aldrich) (*n* = 6 independent experiments). Luciferase assays were performed 48 h after transfection by using the Dual Luciferase Reporter Assay System (Promega, Madison, WI, USA) according to the manufacturer's protocol (*n* = 6 independent experiments). All the analyses were performed in triplicate and data were reported as mean value ± SEM.

### 6.10 Cell Proliferation Assays

TM4 cell proliferation following transfection with mmu‐miR‐214‐3p mimic and siFUS was evaluated by MTT assay (*n* = 6 independent experiments). In brief, cells were plated in 96‐well plates, transfected as detailed above, and 48 h later their growth was evaluated by adding 150 μL of medium with 0.5 mg/mL 3‐(4,5‐dimethylthiazol‐2‐yl)‐2,5‐diphenyl tetrazolium bromide (MTT) to each well; 1 h after incubation at 37°C, the medium was discarded, and the purple formazan crystals produced in the viable cells were solubilized in 100 μL of dimethyl sulfoxide and quantified by measurement of absorbance at 570 nm with a plate reader. Data were expressed as a percentage of cell growth in comparison to the control group (CTRL‐miR or CTRL‐siRNA), which was set to 100, and reported as mean value ± SEM.

### 6.11 Senescence Immunofluorescence Assays

Senescence assay was performed in (i) testes collected from Young (*n* = 6) and Aged (*n* = 6) mice, and (ii) TM4 cells in vitro treated with miR‐214‐3p mimic miRNA or siRNA against FUS (*n* = 6 for each experimental group) using a detection kit specific for β‐galactosidase (C10850, Invitrogen, Milano, Italy) following the manufacturer's instructions. In brief, following fixation, samples were incubated with the fluorescent substrate for β‐galactosidase, having a fluorescence excitation and emission maximum: 490/514 nm, at 37°C without CO_2_ protecting from the light. Nuclei were labeled using DAPI (D9542; Sigma‐Aldrich, Milano, Italy). Samples were analyzed under an optical microscope (Leica DM 5000 B + CTR 5000; Leica Microsystems, Wetzlar, Germany) with a UV lamp, and images were captured by using a Leica DFC320 R2 digital camera.

The intensity of β‐gal fluorescence signal was determined per area with the use of ImageJ Software (version 1.53 g) and adjusted relatively to DAPI fluorescence intensity in the same field. Results were expressed as fold change relative to the reference group and reported as mean ± SEM.

### 6.12 Protein Extraction and Western Blot Analysis

Total protein lysates from Young (*n* = 6) and Aged (*n* = 6) mice testes and TM4 cells in vitro treated with miR‐214‐3p mimic miRNA or siRNA against FUS (*n* = 6 for each experimental group) were obtained by using RIPA extraction buffer [PBS, pH 7.4, 10 mM dithiothreitol, 0.02% sodium azide, 0.1% SDS, 1% NP‐40, 0.5% sodium deoxycholate, and protease inhibitors (10 μg/mL of leupeptin, aprotinin, pepstatin A, chymostatin, and 5 μg/mL of TPCK)]. Sodium dodecyl sulfate–polyacrylamide gel electrophoresis (SDS‐PAGE) (4%–20% Mini‐PROTEAN TGX Precast Protein Gels; 4561094, Bio‐Rad Laboratories, Milano, Italy) was used for protein separation. Then, proteins were transferred to a polyvinylidene difluoride membrane (GE Healthcare, Milano, Italy) at 280 mA for 2.5 h at 4°C. The filters were blocked [5% nonfat milk, 0.25% Tween‐20 in Tris‐buffered saline (TBS, pH 7.6)] and incubated with different primary antibodies, all diluted 1:1000 [FUS antibody (PA5‐52610; Invitrogen, Milano, Italy), SOX9 (sc‐166505; Santa Cruz Biotechnology, Heidelberg, Germany), SOX8 (sc‐374445; Santa Cruz Biotechnology, Heidelberg, Germany), NOTCH2 (sc‐518049; Santa Cruz Biotechnology, Heidelberg, Germany), WT1 (sc‐7385; Santa Cruz Biotechnology, Heidelberg, Germany), DDX4 (MA5‐15565; Invitrogen, Milano, Italy), MAGE‐A4 (PA5‐72867; Invitrogen, Milano, Italy), SCP3 (PA1‐16766; Invitrogen, Milano, Italy), and TUBULIN (ab15246; Abcam, Cambridge, UK)].

### 6.13 Total RNA Preparation

Total RNA was extracted from (i) aliquots of *cauda* SPZ collected from Young and Aged mice (*n* = 6 for each experimental group); (ii) reproductive tissues (testis and epididymis) collected from Young (*n* = 6) and Aged (*n* = 6) mice; (iii) TM4 cells ± siFUS (*n* = 6 for each experimental group); (iv) aliquots of A‐ and B‐SPZ collected from normozoospermic men (*n* = 6 for each experimental group); and (v) aliquots of A‐SPZ collected from Young and Aged men (*n* = 6 for each experimental group) using TRIzol Reagent (Invitrogen Life Technologies, Paisley, UK) following the manufacturer's instructions. In brief, after sample homogenization for 5 min at 20°C and chloroform/mL TRIzol Reagent added to each sample (0.2 mL), the samples were centrifuged at 12000*g* for 15 min at 4°C. Then, total RNA was precipitated by adding isopropyl alcohol (0.5 mL/mL TRIzol Reagent) and 1 μL glycogen (20 mg/mL). Each RNA pellet was washed with 75% ethanol, centrifuged at 7500*g* for 10 min at 4°C, and dissolved in DEPC‐treated water to quantify (ng/μl) and evaluate RNA purity (260/280 and 260/230 ratios) using a NanoDrop 2000 spectrophotometer (Thermo Fisher Scientific, Waltham, MA, USA). Aliquots of RNA (10 μg) were treated with 2 U DNase I (RNase‐free DNase I, Ambion, Thermo Fisher Scientific, Waltham, MA, USA) to remove genomic DNA contamination and stored at –80°C.

### 6.14 RNA Expression Analysis by One‐Step Quantitative RT‐PCR

CircRNA expression analysis was performed in (i) aliquots of *cauda* SPZ collected from Young and Aged mice (*n* = 6 for each experimental group); (ii) reproductive tissues (testis and epididymis) collected from Young (*n* = 6) and Aged (*n* = 6) mice; and (iii) TM4 cells ± siFUS (*n* = 6 for each experimental group in triplicate) by using a One‐Step Evagreen quantitative RT‐qPCR (RT‐qPCR) reaction kit containing a RT‐qPCR enzyme mix and an Evagreen qPCR Mastermix (Applied Biological Materials Inc., Ferndale, WA, USA) and a CFX‐96 Real Time PCR System (Bio‐Rad, Milano, Italy). For each assay, a negative control without RNA and the melting curve analysis of primer pairs were included. For RNA expression analysis, a CFX Manager software (Bio‐Rad, Milano, Italy) was used. Data were normalized against the *Cyclophilin* housekeeping gene. The normalized fold expression (nfe) of circRNAs was calculated by applying the 2^−ΔΔCt^ method. All the results were expressed as a mean value of nfe ± SEM.

### 6.15 RT‐qPCR miRNA Assay

MiR‐214‐3p and the reference U6 snRNA were quantified by miRCURY LNA miRNA SYBR Green PCR System (Qiagen, Germantown, Maryland, USA) according to the manufacturer's protocol. The expression levels of miRNAs were normalized to U6 by using the 2^−ΔCt^ method and reported as fold change (2^−ΔΔCt^) in comparison to control experiments. All the results were expressed as a mean value of nfe ± SEM.

### 6.16 RNA‐Binding Protein Immunoprecipitation Assay

For RIP assay, TM4 cells ± siFUS (*n* = 6 samples for each experimental group) were lysed in RIPA buffer (50 mM Tris‐HCl pH 7.4; 150 mM NaCl; 5 mM EDTA; 1% NP‐40; 0.1% SDS) supplemented with RNase inhibitors (100 U/mL) and protease inhibitors (10 μg/mL of leupeptin, aprotinin, pepstatin A, chymostatin, and 5 μg/mL of TPCK). A concentration of 5 μg of FUS antibody (PA5‐52610; Invitrogen, Milano, Italy) or IgG (12370; Sigma‐Aldrich, Milano, Italy) was incubated with 500 μg of each lysate under rotary agitation at 4°C overnight. Following this, all samples were incubated with Protein A/G PLUS Agarose Beads (sc‐2003; Santa Cruz Biotechnology, Heidelberg, Germany) at 4°C for 4 h. The bead pellets were washed with cold TBS pH 7.6, at 3000 × g for 5 min at 4°C and then resuspended in 500 μL of Trizol Reagent (Invitrogen Life Technologies, Paisley, UK) to isolate RNA, according to the manufacturer's instructions. The immunoprecipitated RNAs were quantified (ng/μl) using a NanoDrop 2000 spectrophotometer (Thermo, Waltham, MA, United States) and used for circAbcb9 RT‐qPCR analysis.

### 6.17 PCR Primer Design

The online tool Primer‐BLAST (http://www.ncbi.nlm.nih.gov/tools/primer‐blast/) was used to design murine and human primers. For circRNA, we designed primers in order to span the back‐splicing junction specific for the circular isoforms. Primer sequences are shown in Table 1.

**TABLE 1:** Primer sequences and annealing temperatures.

Gene primers	Sequences 5′‐3′	Tm (°C)
*circAbcb9* S	TACACAAAGCCTGACGTGGC	58
*circAbcb9* AS	GGGCAAGGCCATCACTGACA	
*circNFAT5* S	AAAAGAGCACTCGTGCCAGA	52
*circNFAT5* AS	TCAGAGAATTGCATAAAATGGGG	
*circAdam10* S	CCTATGTCTTCACAGACCGGG	55
*circAdam10* AS	TGGGGATAGTCTGAAGGTGC	
*circQRICH1* S	TGGGAATGTAAGCAGCTTGGG	56
*circQRICH1* AS	TGACAATCCACCTGAGGCT	
*circINPP5B* S	CCTCCTGCCAGTGAGAAGTG	58
*circINPP5B* AS	TCAGCTGAGTGATGTTCTTTCCT	
*circSipa1l2* S	ACGACTGCTACGGATTCCAC	58
*circSipa1l2* AS	CAGCGTCGACACATGGAACA	
*Cyclophilin‐A S*	TGGTCTTTGGGAAGGTGAAAG	52
*Cyclophilin‐A* AS	TGTCCACAGTCGGAAATGGT	
*Fus* S	GGTGGTGGAGGCAACTATGG	56
*Fus* AS	GTCACTTCCGCCCATGCCGC	
*Hsa‐circAbcb9* S	CTGCTCTCCTACACCAAGCC	58
*Hsa‐circAbcb9* AS	GGCCCAGAGCTGCCACGAT	
*Gapdh* S	TGCACCACCAACTGCTTAGC	58
*Gapdh* AS	GGCATGGACTGTGGTCATGAG	

### 6.18 Functional Annotation for circRNA/miRNA and Target miRNA Interaction

The circRNA/miRNA interaction for circABCB9, circNFAT5, circAdam10, circQRICH1, circINPP5B, circSipa1l2, and has‐circABCB9 was predicted with Arraystar's miRNA target prediction software and circATLAS 3.0 (http://circatlas.biols.ac.cn/). The miRNA targets were obtained by Diana TarBase 8.0 (http://www.microrna.gr/tarbase); Bisogenet plug‐in of Cytoscape (www.cytoscape.org) was used to build circRNA/miRNA/Target networks (ceRNETs).

### 6.19 Statistical Analysis

Shapiro‐Wilk test was used to assess data normality and to confirm the normal distribution of data. Following data confirmation, the Student's *t*‐test (for two independent group comparisons) was used to identify groups having different means. Differences with *p* < 0.05 were considered statistically significant; all data were expressed as the mean ± SEM. For molecular investigations and cell counting assays, triplicate of at least six different samples for each experimental group were considered statistically significant.

## Author Contributions

F.M., N.M., V.G.M., and T.C. performed the experiments. F.M., N.M., and T.C. wrote the manuscript. F.M., N.M., G.M., C.C., and M.M. analyzed the data. G.M., S.F., G.C., N.P., and R.C. designed, supervised, and revised the manuscript. All authors read and approved the final manuscript.

## Disclosure

CircAbcb9‐ceRNET regulates Sertoli cell survivor. The aging induces a shutdown of circAbcb9‐dependent network associated with a prominent increase in Sertoli cell senescence. Consequently, the impaired spermatogenesis leads to defective spermatozoa having low circAbcb9 content. A new intriguing role of a circRNA as hallmark of age‐dependent fertility decline is suggested.

## Ethics Statement

The animal study was conducted according to the National Research Council's publication Guide for Care and Use of Laboratory Animals (National Institutes of Health Guide) and approved by the Italian Ministry of Education and the Italian Ministry of Health with authorization n. 405/2021‐PR issued on June 7, 2021. The ethics committee of Azienda Sanitaria Locale (ASL) Caserta, Regione Campania (ASL CE Prot. N. 1217885/DIR. GE) reviewed and approved the study involving human participants. These latter provided written informed consent in accordance with the Declaration of Helsinki.

## Conflicts of Interest

The authors declare no conflicts of interest.

## Supporting information


**Figure S1.** Validation of circAbcb9 in human spermatozoa. (A) The hsa‐circAbcb9/miR‐214‐3p network was built using Cytoscape. Hexagonal and rectangular symbols represent hsa‐circAbcb9 and hsa‐miR‐214‐3p, respectively. The arrow indicates the tethering activity of hsa‐circAbcb9 towardhsa‐miR‐214‐3p, while the dotted arrow indicates the pathways downstream of the miRNA. (B–C) Expression analysis of hsa‐circAbcb9 in (B) A‐ and B‐SPZ of normozoospermic men (*n* = 6 in triplicate) and in (C) A‐SPZ collected from Young and Aged men (*n* = 6 in triplicate). RT‐qPCR data were normalized using *Gapdh*, expressed as fold expression (nfe), and reported as mean value ± S.E.M; **p < 0.01; *p < 0.05.

## Data Availability

The data that support the findings of this study are available on request from the corresponding author. The data are not publicly available due to privacy or ethical restrictions.
