# Supplementary figures and images for: Deciphering the Contribution of Circular RNAs to Age‐Related Decline in Sertoli Cell Survivor
Source: Aging Cell. 2025 Apr 18;24(6):e70023. doi: 10.1111/acel.70023 (PMC12151912; doi:10.1111/acel.70023)

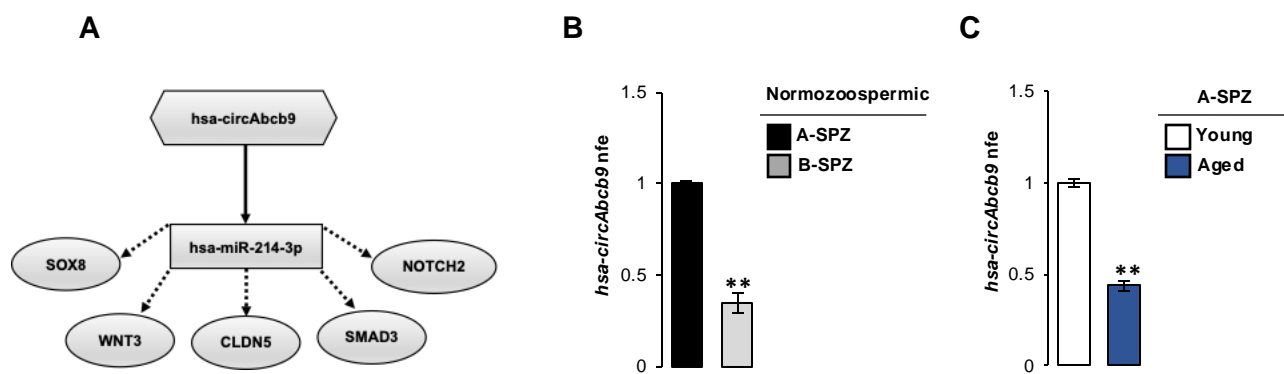

SUPPLEMENTAL FIGURE 1

Supplement: Supplementary file 1 — Figure S1. Validation of circAbcb9 in human spermatozoa. (A) The hsa‐circAbcb9/miR‐214‐3p network was built using Cytoscape. Hexagonal and rectangular symbols represent hsa‐circAbcb9 and hsa‐miR‐214‐3p, respectively. The arrow indicates the tethering activity of hsa‐circAbcb9 towardhsa‐miR‐214‐3p, while the dotted arrow indicates the pathways downstream of the miRNA. (B–C) Expression analysis of hsa‐circAbcb9 in (B) A‐ and B‐SPZ of normozoospermic men (n = 6 in triplicate) and in (C) A‐SPZ collected from Young and Aged men (n = 6 in triplicate). RT‐qPCR data were normalized using Gapdh, expressed as fold expression (nfe), and reported as mean value ± S.E.M; **p < 0.01; *p < 0.05. [file ACEL-24-e70023-s001.pdf]
